# Monitoring of Biological Changes in Electromechanical Reshaping of Cartilage Using Imaging Modalities

**DOI:** 10.1155/2016/7089017

**Published:** 2016-12-08

**Authors:** Seok Jin Hong, Minseok Lee, Connie J. Oh, Sehwan Kim

**Affiliations:** ^1^Department of Otorhinolaryngology-Head and Neck Surgery, Hallym University, Dongtan Sacred Heart Hospital, 7 Keunjaebong-gil, Hwaseong-si, Gyeonggi-do 18450, Republic of Korea; ^2^Department of Biomedical Engineering, College of Medicine, Dankook University, 119 Dandae-ro, Dongnam-gu, Cheonan, Chungnam 31116, Republic of Korea; ^3^Beckman Laser Institute and Medical Clinic, University of California, Irvine, 1002 Health Sciences Rd., Irvine, CA 92612, USA

## Abstract

Electromechanical reshaping (EMR) is a promising surgical technique used to reshape cartilage by direct current and mechanical deformation. It causes local stress relaxation and permanent alterations in the shape of cartilage. The major advantages of EMR are its minimally invasive nature and nonthermal electrochemical mechanism of action. The purpose of this study is to validate that EMR does not cause thermal damage and to observe structural changes in post-EMR cartilage using several imaging modalities. Three imaging modality metrics were used to validate the performance of EMR by identifying structural deformation during cartilage reshaping: infrared thermography was used to sense the temperature of the flat cartilages (16.7°C at 6 V), optical coherence tomography (OCT) was used to examine the change in the cartilage by gauging deformation in the tissue matrix during EMR, and scanning electron microscopy (SEM) was used to show that EMR-treated cartilage is irregularly arranged and the thickness of collagen fibers varies, which affects the change in shape of the cartilage. In conclusion, the three imaging modalities reveal the nonthermal and electromechanical mechanisms of EMR and demonstrate that use of an EMR device is feasible for reshaping cartilage in a minimally invasive manner.

## 1. Introduction

Cartilage forms the framework of the upper airway and the structural aesthetic features of the face, and reshaping living tissue without the need for incisions or sutures has been an elusive goal of surgeons for over a century. Carving, morselizing, scoring, or suturing tissue are conventional surgical techniques used to reshape cartilage that act by balancing the forces that resist deformation of the cartilage. Although such techniques are considered the gold standard for reshaping cartilage, they are associated with variable results depending on the preferences and skills of individual surgeons [1–3].

Alternative and minimally invasive methods to reshape living tissue have been under development for more than a decade. Laser cartilage reshaping (LCR) [4–6] and radio frequency cartilage reshaping [7–9] achieve permanent shape changes by a thermoforming technique of which disadvantage is tissue damage due to uncontrolled heat diffusion. Although laser reshaping of nasal septum is possible without dramatic cell damage, the window for energy application without tissue damage is narrow. Without the knowledge of the cartilage thickness and specific conditions, it is difficult to determine the application energy and time for LCR that will not cause damage [4]. Beyond this energy application window, the LCR will cause heat induced cell damage [5, 6]. In contrast, Electromechanical Reshaping (EMR) can reshape cartilage using electroforming and exerts its effects in a nonthermal and minimally invasive fashion [10–13]. Thus, EMR has received increasing attention as an alternative to traditional cut and suture surgical techniques in reconstructive and aesthetic surgery.

EMR is used to alter the shape of cartilage by initiating oxidation-reduction reactions in the vicinity of high-stress concentrations of mechanically deformed cartilage specimens. In more detail, the mechanism of EMR is as follows: first, cartilage is mechanically deformed or straightened into the desired shape. Then, low-level dc voltage applied by electrodes generates a direct currents in the milliampere range, which leads to a series of redox reactions at the tissue-electrode interface. It is known that temperature elevation during EMR is negligible, suggesting that shape change occurs without any contribution from resistive heating [11]. However, it was noted that cell damage may occur due to mechanical trauma and electrochemical effect [12].

Nonetheless, EMR is a relatively simple, noninvasive, electroforming, and portable technology. The potential clinical applications of the EMR include procedures essential to numerous reconstructive and aesthetic operations, including shape modification of cartilage tissue in the face, neck, and upper airways. Specifically, EMR may be used for correction deviations of the nasal septum, correction deformities and defects of the external ear, reconstruction of congenital malformations of the external ear (otoplasty), and treatment of postintubation stenosis in trachea and larynx.

Although previous studies on EMR have revealed its chemical mechanisms, voltage-current dosimetry, minimally invasive nature, and electroforming characteristics, it remains necessary to investigate the structural changes that take place at the tissue level following EMR. In this study, we scrutinize the efficacy of structural change in the absence of a thermoforming effect during EMR using three imaging modalities, scanning electron microscopy (SEM), optical coherence tomography (OCT), and infrared thermography.

## 2. Materials and Methods 

### 2.1. EMR System Equipped with Multiple Platinum Needles

A multiple platinum needle-based EMR system was developed for minimally invasive surgery in this study. The system is handheld, so it is appropriate for in-office or bedside operations, in contrast to previous, bulky bench-top EMR machines [11, 13].

**Figure 1:**
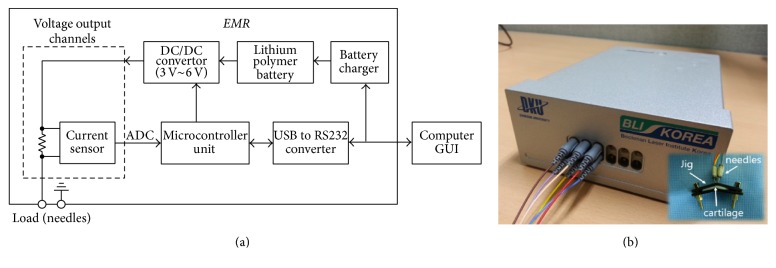
Electromechanical Cartilage Reshaping System: (a) system block diagram; (b) photo of EMR system for office or bedside operations.

Figure 1 shows a system block diagram and a photo of the multiple needle electrode-based EMR system. The EMR system has six arrays of platinum needle electrodes, of which each array is composed of a positive-negative pair. Therefore, a geometric configuration of multiple platinum needle electrode pairs can cover a wide area of cartilage in a minimally invasive manner. The output voltage of the positive needles can be programmed between 3 V and 6 V in 1 V steps. During voltage application, the current flow can be measured by current sensors located near each positive electrode. The current sensor is a type of current shunt monitor, which can sense the voltage drop across a shunt-resistor using an analogue-to-digital converter (ADC).

The sensed voltage drops are translated into an amount of electrical current by the software in the controller and displayed as a chart in the computer's graphical user interface (GUI). Finally, the GUI delineates the amount of electric current according to the voltage and electrical energy applied during cartilage reshaping for each platinum needle array.

### 2.2. Tissue Preparation and EMR Protocol

Fresh rabbit nasal septal cartilage was extracted from the crania of New Zealand White Rabbits. The cartilage was cut into 23 mm × 6.0 mm slices (width × height) with three different thicknesses, 0.5, 1, and 1.5 mm, using a sharp blade. Four cartilage specimens were used to create the cartilage thermograph, while five cartilage specimens were used per group to sense the structural changes.

During temperature measurement testing for the thermograph, three pairs of electrodes were inserted into the middle of the rabbit nasal septal cartilage and the temperature was measured for six minutes at applied voltages of 3 V, 4 V, 5 V, and 6 V by the infrared FLIR camera. The camera was focused on the center of the three positive needles to record temperature variation in the cartilage during voltage application.

To examine the structural changes in cartilage, the three arrays of positive-negative platinum needle electrodes were separated by a distance of 6 mm. The platinum needle electrodes were positioned 2 mm apart. The specimen was mechanically deformed 90 degrees and fixed to a plastic jig. Platinum needle-based electrodes were penetrated into the cartilage through the jig. Three negative needles and three positive needles were placed parallel to one another across the bend. The electrodes were connected to the EMR device through the female needle ports, as shown in Figure 1(b). The voltage and exposure duration can be set using a touch screen device; 3, 4, 5, and 6 volts and 1, 3, 5, and 6 minutes of exposure time were used for the analysis.

After EMR, the needles were removed, and the specimen was placed in a phosphate buffered solution at a pH of 7.4 and allowed to rehydrate for 15 minutes. After rehydration, the specimen was allowed to sit in the open air for one additional minute before optical coherence tomography (OCT) and scanning electron microscopy (SEM) were performed.

### 2.3. Imaging Modalities for Investigation of EMR Mechanism

To thermally image the cartilage, a layout of three pairs of needles 2 mm apart from each other was inserted into the cartilage. The row of positive needles served as the anode; the other served as the cathode. Constant voltage was applied at 3, 4, 5, or 6 V for 6 min while the temperature was monitored using an FLIR camera (FLIR SC 660).

Optical coherence tomography (OCT) was employed to examine tissue layer-level changes in the cartilage during EMR. Two platinum needle electrodes were inserted into fixed rectangular rabbit nasal septal cartilage specimens. The spectral domain OCT probe was then positioned above the section of cartilage in which the anode needle was inserted. A constant voltage of 6 V was applied for 3 minutes, and images were obtained (8 frames/second). Specimens were captured three times with OCT (before and immediately after EMR and after rehydration with PBS for 15 minutes). OCT is a useful imaging modality to monitor the difference in morphological changes between the anode and the cathode needles insertion sites during electromechanical reshaping. As gold nanoparticles are potential contrast agents for OCT imaging, they provide contrasting cartilage layer borders. Gold nanoparticles were used only during the OCT measurement and not during EMR treatment.

Control- and EMR-treated (6 V, 5 min) specimens were fixed for 24 hr, dehydrated in an ethanol series (30%, 50%, 70%, 90%, 100%, and filtered 100% ethanol), and dried using tetramethylsilane in air. The specimens were coated using a 30 nm layer of Au-Pd, mounted onto Al stubs using carbon tape, and imaged using a scanning electron microscope (JEOL JSM 6330F) operated at an accelerating voltage of 3 keV. We used ×500, ×1000, and ×2000 magnification for SEM.

## 3. Results

To define the mechanisms of action of EMR, we conducted experiments using rabbit nasal septal cartilage. Three metrics were used to determine the performance and mechanism of EMR: thermograph of cartilage during voltage application, which was used to analyze electroforming features, optical coherence tomography (OCT), which was used to identify the changes in layers of cartilage during EMR, and scanning electron microscopy (SEM), which was used to monitor fiber-level changes in the tissue matrix.

### 3.1. Image Modality I: Infrared Thermography

**Figure 2:**
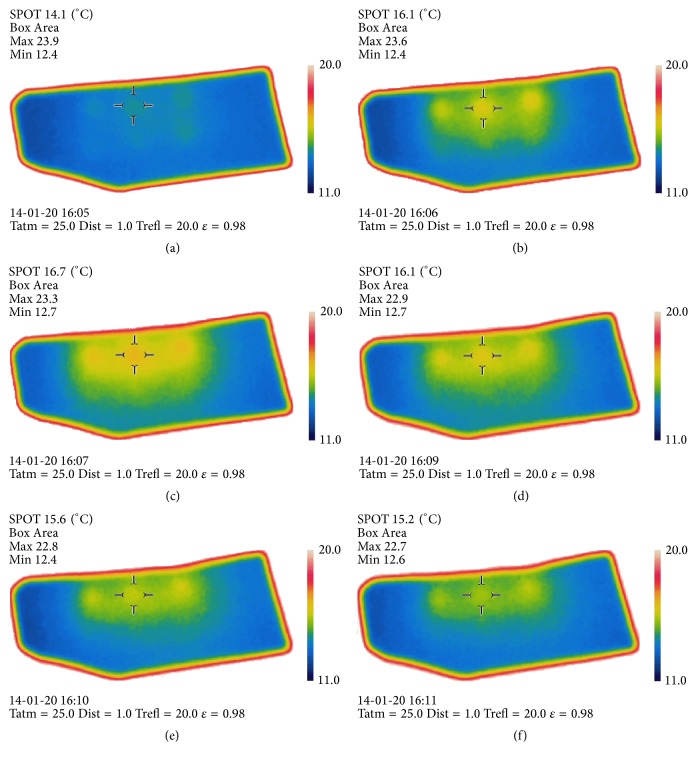
Infrared thermographs of temperature during EMR with an applied voltage of 6 V: (a) baseline (0 sec); (b) after 1 min; (c) after 2 min; (d) after 4 min; (e) after 5 min; (f) after 6 min.

In order to verify that heat is not a main factor in reshaping of cartilage during EMR, we conducted an experiment using an infrared camera (FLIR SC 660). The peak temperature was measured during application of 3, 4, 5, and 6 V for 6 min. The anode needles generated more heat than did the cathode electrodes. According to Figure 2, temperature increased with increasing voltage. With the application of 6 V to rabbit nasal septal cartilage, the temperature rose from an initial temperature of 14.1°C to reach a peak temperature of 16.7°C in 2 minutes at the positive needles. After reaching the peak temperature of 16.7°C, the temperature of the cartilage began to decrease. In addition, an increase in applied voltage causes a reduction in time taken to reach the peak temperature.

### 3.2. Image Modality II: Optical Coherence Tomography

**Figure 3:**
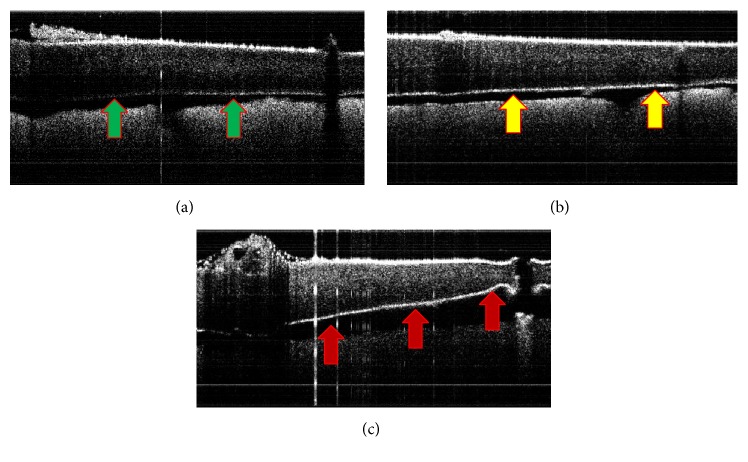
Optical coherence tomography findings of EMR-treated cartilage: (a) control specimens, baseline image without gold nanoparticles (green arrows); (b) baseline image with gold nanoparticles (yellow arrows); (c) after application of 6 V for 3 mins. Image of EMR-treated cartilage with gold nanoparticles (red arrows).

Images taken by optical coherence tomography (OCT) are shown in Figure 3. We used gold nanoparticles to clearly investigate the efficacy of EMR in cartilage reshaping at the tissue layer level. At baseline, comparison of images with or without gold nanoparticles indicated that the surface of cartilage lined with gold nanoparticles could be clearly identified (Figures 3(a) and 3(b)). Shrinkage was observed on OCT images after EMR treatment (Figure 3(c)), which did not recover after immersion of samples in PBS for 15 minutes. The cartilage shrinkage was uneven on the anode and the cathode region.

### 3.3. Image Modality III: Scanning Electron Microscopy

**Figure 4:**
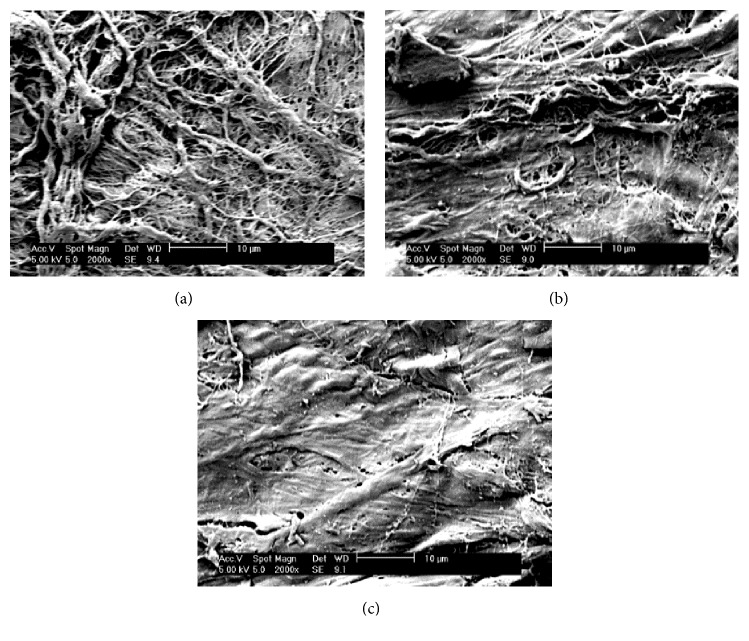
Scanning electron microscopy images of rabbit nasal septal cartilage specimens. Images (a), (b), and (c) were taken at ×2000 magnification. (a) Control specimens show organized, layered, thick collagen fibers. (b) EMR-treated (6 V, 5 mins) specimens at the positive electrode needle insertion site (anode). (c) EMR-treated specimens (6 V, 5 mins) at the negative electrode needle insertion site (cathode). EMR-treated cartilage obtained from the needle electrodes insertion sites shows irregularly arranged collagen fibers that vary in thickness. The specimens at the negative needle electrode show more loosely connected, damaged, irregular fibers compared with those at the positive needle electrode.

The images taken with scanning electron microscopy (SEM) are shown in Figure 4. SEM images of rabbit nasal septal cartilage show fine, organized, fibrous collagen fibers (Figure 4(a)). There were significant differences in irregularities and loss of connection between the control group and the EMR-treated groups. Images of EMR-treated rabbit septal cartilage of the positive needle electrode demonstrate less well-organized, irregularly arranged collagen fibers as compared with untreated cartilage (Figure 4(b)). SEM images of the negative needle electrode show fibers that appear melted and damaged compared with those at the positive pole (Figure 4(c)).

## 4. Discussion

One of the major challenges in cartilage engineering is the development of techniques to create desired aesthetic and functional structures with less morbidity than traditional surgical approaches. There have been previous studies on laser cartilage reshaping (LCR) [4–6] and radiofrequency cartilage reshaping [7–9], which have been shown to effectively reshape cartilage in living tissue. However, these thermal methods may lead to tissue injury due to uncontrolled heat diffusion, and it is necessary to find a compromise between cartilage shape change and cellular damage. The critical temperature for stress relaxation in septal cartilage occurs between 60 and 75°C [6, 14]. The main mechanism of LCR is heat-associated stress relaxation, and heat causes physical changes in tissues that may not only result in reshaping but also injure chondrocytes. Radiofrequency reshaping is a temperature-dependent process as well, in which surface temperatures reach a maximum of 88.6°C in less than 5 seconds [8].

EMR is a promising technique for cartilage engineering that may be a good alternative to traditional surgery. EMR has a distinct benefit in that it avoids heat-associated protein denaturation and cell death. Protsenko et al. previously reported that EMR is effective in accelerating stress relaxation and reshaping cartilage, with approximately 20 percent of chondrocytes surviving after 2–4 weeks following EMR procedures that produced clinically significant reshaping [11, 12]. Unlike thermal reshaping methods, EMR does not generate enough heat to generate high temperatures in the tissue. We sought to assess the feasibility of EMR for cartilage engineering with thermography and imaging modalities. The results of thermography show that the EMR caused the cartilage temperature to rise merely by 2.6°C (14.1°C to 16.7°C) with the application of 6 V on the flat cartilage at the positive needle insertion site. This result suggests that EMR is not a thermoforming but an electroforming technique.

Optical coherence tomography (OCT) is a noninvasive, high-speed image modality. Different materials have been used as contrast agents in OCT imaging of tissue structures; among them, gold nanoparticles are biocompatible, easily synthesized, and nontoxic. Also, gold nanoparticles are easy to manipulate owing to their optical properties. They can be functionalized to target specific cellular biomarkers and have been used in cancer imaging [15].

In our OCT findings, there was layer shrinkage observed during EMR, and the shrinkage did not recover after specimens were immerged in PBS for 15 minutes. Besides, the extent of cartilage shrinkage at the positive needle electrode differed from that of the negative needle electrode on the EMR-treated cartilage. This finding indicates that EMR induces an unbalance shape change between the positive and negative needle electrodes. This varying extent of cartilage shrinkage suggests that the pH changes at the anode and cathode sites are responsible for the varying extent and type of injury differs from anode and the cathode [16]. With the OCT results, we also confirmed that gold nanoparticles are useful for the detection and accurate identification of the structural changes that take place during EMR.

Although the precise mechanisms of EMR are unknown, Ho et al. proposed several possible mechanisms [17]. One of the hypotheses is a loss of water through hydrolysis. Hydrogen ions are reduced to form hydrogen gas at the cathodes, while hydroxide ions are oxidized to produce oxygen gas at the anode. Another possible mechanism of EMR is electrophoresis. Cartilage is composed of organized collagen fibrils and a proteoglycan matrix filled with Na^+^, K^+^, Cl^−^, and other ions. The movement of such ions between the needles electrodes during EMR may redistribute the negative charges in the extracellular matrix to induce permanent shape change [12].

The SEM images of control, untreated cartilage revealed organized, layered, thick collagen fibers. The SEM images of EMR-treated rabbit septal cartilage at the positive pole demonstrated less well-organized, irregularly arranged collagen fibers. SEM images of the negative pole show melted and damaged fibers compared with those at the positive pole. Our findings reveal that during EMR the electric field produces an electrochemical reaction at the electrodes, negatively charged proteoglycan molecules may move to the positive pole, and chondrocytes near the electrodes may be damaged via electrophoresis and oxidation-reduction reactions.

## 5. Conclusions

In this study, we verified the electroforming feature of the EMR using infrared thermography and explored the structural changes in the tissue matrix of cartilage using optical coherence tomography (OCT) and scanning electron microscopy (SEM).

LCR or radiofrequency reshaping techniques require generation of heat at a maximum of 75°C or 88.6°C, respectively, to reshape the cartilage, while the EMR caused the peak temperature up to 16.7°C. This result reveals that EMR is not a thermoforming but an electroforming technique. Therefore, EMR is associated with localized cell injury. In addition, the difference in the cartilage shrinkage between the anode and the cathode during EMR was identified using OCT. Gold nanoparticles were helpful in enhancing the clarity of the layer changes that take place during EMR. SEM of rabbit nasal septal cartilage revealed structural changes in the collagen fibers after EMR treatment.

The use of thermography for temperature detection during EMR and the use of imaging modalities to visualize the effect of EMR on the morphological changes in the tissue will add to our understanding of electrochemical reactions and potentially help to optimize EMR dosimetry. In the near future, as the knowledge of EMR builds up, EMR may evolve from a promising theoretical method to a feasible office-based or bedside service care technique.
